# Surviving in steep terrain: a lab-to-field assessment of locomotor costs for wild mountain lions (*Puma concolor*)

**DOI:** 10.1186/s40462-020-00215-9

**Published:** 2020-08-08

**Authors:** Carolyn E. Dunford, Nikki J. Marks, Christopher C. Wilmers, Caleb M. Bryce, Barry Nickel, Lisa L. Wolfe, D. Michael Scantlebury, Terrie M. Williams

**Affiliations:** 1 School of Biological Sciences, Institute of Global Food Security, Queen’s University of Belfast, 19 Chlorine Gardens, Belfast, BT9 5DL Northern Ireland; 2 Center for Integrated Spatial Research, Environmental Studies Department, University of California- Santa Cruz, Santa Cruz, CA 95064 USA; 3 Botswana Predator Conservation Trust, Maun, Botswana; 4 Colorado Division of Parks and Wildlife, Wildlife Health Program, 4330 Laporte Avenue, Fort Collins, CO 80521-2153 USA; 5 Department of Ecology and Evolutionary Biology, Coastal Biology Building, 130 McAllister Way, University of California- Santa Cruz, Santa Cruz, CA 95060 USA

**Keywords:** Energy expenditure, Energetics, Oxygen consumption, Indirect calorimetry, Locomotion, Incline, Carnivore, Accelerometer, Global change

## Abstract

**Background:**

Under current scenarios of climate change and habitat loss, many wild animals, especially large predators, are moving into novel energetically challenging environments. Consequently, changes in terrain associated with such moves may heighten energetic costs and effect the decline of populations in new localities.

**Methods:**

To examine locomotor costs of a large carnivorous mammal moving in mountainous habitats, the oxygen consumption of captive pumas (*Puma concolor*) was measured during treadmill locomotion on level and incline (6.8°) surfaces. These data were used to predict energetic costs of locomotor behaviours of free-ranging pumas equipped with GPS/accelerometer collars in California’s Santa Cruz Mountains.

**Results:**

Incline walking resulted in a 42.0% ± 7.2 SEM increase in the costs of transport compared to level performance. Pumas negotiated steep terrain by traversing across hillsides (mean hill incline 17.2° ± 0.3 SEM; mean path incline 7.3° ± 0.1 SEM). Pumas also walked more slowly up steeper paths, thereby minimizing the energetic impact of vertical terrains. Estimated daily energy expenditure (DEE) based on GPS-derived speeds of free-ranging pumas was 18.3 MJ day^− 1^ ± 0.2 SEM. Calculations show that a 20 degree increase in mean steepness of the terrain would increase puma DEE by less than 1% as they only spend a small proportion (10%) of their day travelling. They also avoided elevated costs by utilizing slower speeds and shallower path angles.

**Conclusions:**

While many factors influence survival in novel habitats, we illustrate the importance of behaviours which reduce locomotor costs when traversing new, energetically challenging environments, and demonstrate that these behaviours are utilised by pumas in the wild.

## Background

The ability of individual animals and animal populations to survive depends, in part, on their ability to balance energy expenditure with energy acquisition within a stochastic environment [1]. In recent years, the behavioural and physiological repertoire of large predatory mammals to maintain energy balance has been challenged by the magnitude and rapidity of environmental perturbations associated with climate change and habitat loss [2, 3]. One of the most obvious and energetically costly responses necessary to meet the comparatively high-resource demands of a carnivorous lifestyle [4], is associated with an increase in distance moved in search of food, often requiring travel into and within new habitats [5].

Because locomotory activities often account for a large proportion of a mammal’s daily energy expenditure (DEE) (see [6–8] for a discussion of transport costs), energy balance can be compromised in highly mobile carnivores as daily activity increases [9, 10]. Changes in body orientation, gait, speed, and manoeuvring have been linked to environmental factors [11, 12] and subsequently to increased overall energy expenditure. It follows that locomotor responses that promote maximum energetic efficiency while minimizing the costs of transport when transiting though difficult terrains are considered beneficial [13].

Recently, there has been growing interest in using accelerometer-based technology to record simultaneous behavioural and energetic responses of animals transiting different habitats (for example [10, 14, 15]). To date, accelerometers have been used primarily to predict energetic costs associated with various body movements, changes of direction, and gaits [15–17]. By comparison, few studies have specifically used this technology to assess the energetic consequences of travelling in different terrains [18]. Higher energetic costs are presumed to occur in more challenging environments such as thick vegetation and sandy soils [19, 20]. Similarly, locomotion on inclines can instigate an increase in energy expenditure compared to that incurred on level ground [21]. Consequently, large animals, such as elephants are suggested to avoid shallow incline slopes when travelling to minimise energetic costs [22–24]. Despite this, some mammals appear to select what would be considered energetically disadvantageous habitats (i.e., mountain ranges). Here the puma (*Puma concolor*) provides a unique opportunity to evaluate how energetic balance and locomotor efficiency can be maintained in a large predator that moves across a complex landscape.

Pumas frequent a wide range of habitats in North and South America [25], which includes mountainous, challenging terrains. This medium-sized (41–68 kg) felid has large home ranges (up to 723 km^2^) [26]. However, they are considered to be specialist ‘stalk-and-pounce’ predators [26, 27] and it has been suggested that pumas may be energetically constrained by an inability to increase energy demands due to their low aerobic scope [28]. This physiological limitation coupled with habitat loss and fragmentation will likely have an impact on the numbers and distribution of pumas as they are increasingly pressured to move into novel environments with unpredictable energetic demands [29]. If wild pumas are constrained by a low aerobic scope, pumas in steep terrains would have to travel slowly and on shallow inclines to avoid exceeding their lactate threshold [28, 30]. Alternatively, pumas could spend the majority of their time at rest in order to decrease the effect of terrain on overall energy expenditure and/or recover from high exercise performance levels.

To determine how locomotory strategies used by pumas may mitigate the potentially high costs of living in mountainous habitats, we investigated one obvious challenge; how steep terrain influences DEE. This was achieved by quantifying the energetic costs of walking on different gradients and at different speeds in the laboratory, and then monitoring how wild pumas living in the Santa Cruz Mountains (California) accrue or avoid these costs. For wild pumas, we assessed daily behaviours and assigned an energetic cost to each based on the laboratory measurements. The wild puma behaviour was identified from GPS and accelerometer data that were calibrated through observations of captive pumas, and the gradient and speed of their locomotion was assessed from the difference in altitude and distance between GPS points. Using this approach, we found that extraordinary metabolic demands due to steep terrains were circumvented by, 1) spending a low proportion of the day actively moving, 2) avoiding steep inclines by horizontally traversing hillsides, and 3) walking more slowly on inclines.

## Methods

In a lab-to-field protocol, we used trained pumas to calibrate SMART collars (described below) that were subsequently deployed on wild counterparts in the Santa Cruz Mountains (CA). Details of the instrumentation and the development of behavioural and energetic signatures from accelerometers incorporated into the SMART collars have been reported previously [14], and are summarized here.

### Laboratory energetics

#### Animals

Three adult pumas (n = two males, one female, body mass = 65.7±4.4 kg), that originated as cubs from the wild, were hand-reared and trained using operant conditioning methods over 10 months to walk in a metabolic chamber mounted on a motorized treadmill. The animals lived in a natural habitat (50 m × 60 m pens) connected to an indoor facility (Foothills Wildlife Research Facility – Colorado Division of Parks and Wildlife, Fort Collins, CO, USA) and fed wild game carcass 2–3 times weekly and diced game meat daily when training. Energetic and kinematic tests were conducted during both winter and summer months. Ambient air temperature during the treadmill tests, T_air_, ranged from 11.1 °C (winter) to 34.0 °C (summer).

#### Open-flow respirometry

Pumas rested and walked on a variable-speed, motorized treadmill (PAWWWS Treadmills, Carson, IA, USA) with a Plexiglas and steel framed metabolic chamber (62 cm W × 92 cm H × 200 cm L) mounted on top. Pumas were post-absorptive following an overnight fast, and tested once per day. Each session began with a pre-exercise resting measurement on sedentary animals followed by steady-state walking or running at a single speed. Total time in the respirometer was approximately 20–30 min with each steady-state trial period lasting a minimum of 10 min. Two pumas voluntarily walked on a level and a moderate incline (6.8^o^) surface at speeds up to 2 m s^− 1^ and 1.1 m s^− 1^, respectively. One puma was included in resting measurements only. Incline angle was selected to provide an additional energetic challenge to the pumas while ensuring that the animals could complete the 10 min test period.

The rate of oxygen consumption ($\dot{V}{\mathrm{O}}_2$) was determined using the protocols of Williams et al. [14] (and see Supplementary Information). The rate of oxygen consumption $\dot{V}{\mathrm{O}}_2$ was determined using the protocols of Williams et al. [14] (see also Supplementary Information). The cost of transport (COT) was calculated by dividing $\dot{V}{\mathrm{O}}_2$ by treadmill speed during each test. Puma $\dot{V}{\mathrm{O}}_2$ was calibrated with overall dynamic body acceleration (ODBA, see below) from the output of accelerometers on the SMART collar by Williams et al. [14].

#### Behavioural signature library

Accelerometer signals from the collars were related to specific behaviours by recording daily movements of collared, captive pumas. A running diary for each animal was recorded by observers with stopwatches and supplemented with video sequences (30 fps, Sony HDR-CX240, Sony Corp., USA, described in detail in ref. [14]) (see also Supplementary Information). Behaviour categories that were considered to be important for the wild pumas were locomotion, non-mobile activities (e.g. eating and grooming), and resting. These are a subset of the behaviour categories detailed in Williams et al. [14], in which the focal behaviours were uniquely identified. These same categories were then used to create a decision tree which also incorporated GPS-derived speed to confirm when the pumas were travelling (Supplementary Fig. 1).

**Fig. 1:**
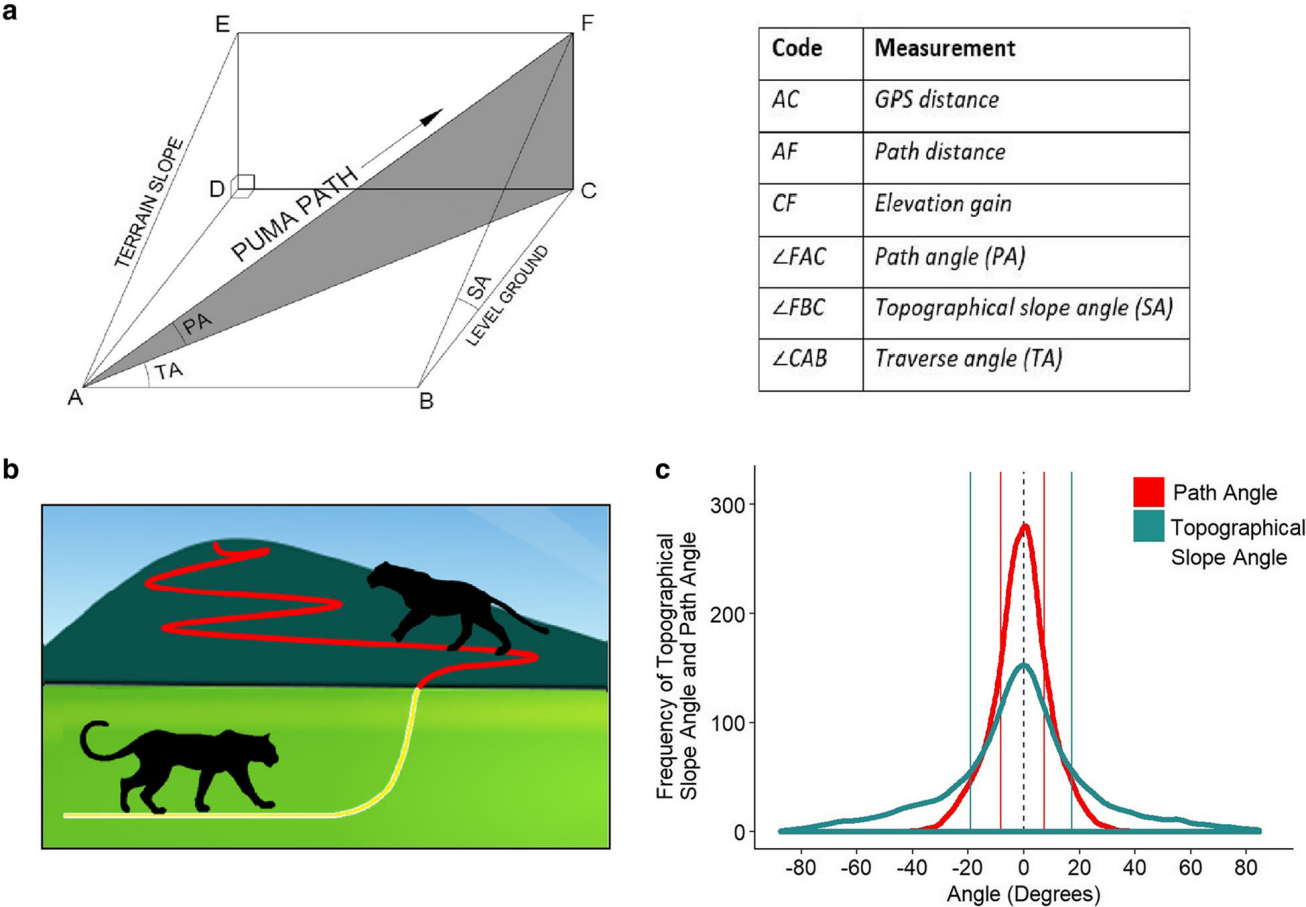
**a** “Cheese wedge” model of slope climbed by pumas. Topographical slope angle (SA) describes the steepness of the hill the puma is standing on. Path angle (PA) is the steepness of the path taken by the puma as it walks up the hill (PA ≤ SA). Traverse angle (TA) is the horizontal angle that the puma walks up the hill. The horizontal GPS distance travelled is the distance between A and C. The path distance the puma travelled, is the distance between points A and F. The elevation gain is the distance between C and F. **b** A schematic showing proposed travelling methods by pumas for different slopes. The yellow path is locomotion on the level ground (direct routes) and the red path is traversing inclining terrain. **c** Frequency of topographical slope angles encountered (green), and path angles chosen by pumas (red). Mean (solid line) shown for incline and decline for each. The dashed line is zero degrees

## Field research

### Study animals and site

Four wild adult male pumas were fitted with a SMART neck collar consisting of an integrated GPS/accelerometer logger (model GPS Plus, Vectronics Aerospace, Germany; total collar mass = 480 g). The animals were captured using either trailing hounds or cage traps, as part of a larger study on puma ecology. Pumas were anesthetized with Telazol® (Fort Dodge Laboratories, Fort Dodge, IA, USA), sexed, weighed, measured for length, and fitted with an identifying ear tag and collar. Males were selected because they are more mobile than females [31]. GPS loggers recorded one fix every five minutes and tri-axial accelerometers recorded at 32 Hz [32] for the duration of the study period. Pumas were monitored for approximately 2 months each (58.25 ± 2.56 days) in 2016; two starting in May, one in October, and one in December (see Supplementary Table 2). The study area, the Santa Cruz Mountains (California, USA), ranges from sea level to 1155 m elevation and has a Mediterranean climate with a diverse landscape of urban developments as well as undisturbed native vegetation (see [31]).

### Landscape and path descriptions

**Fig. 2:**
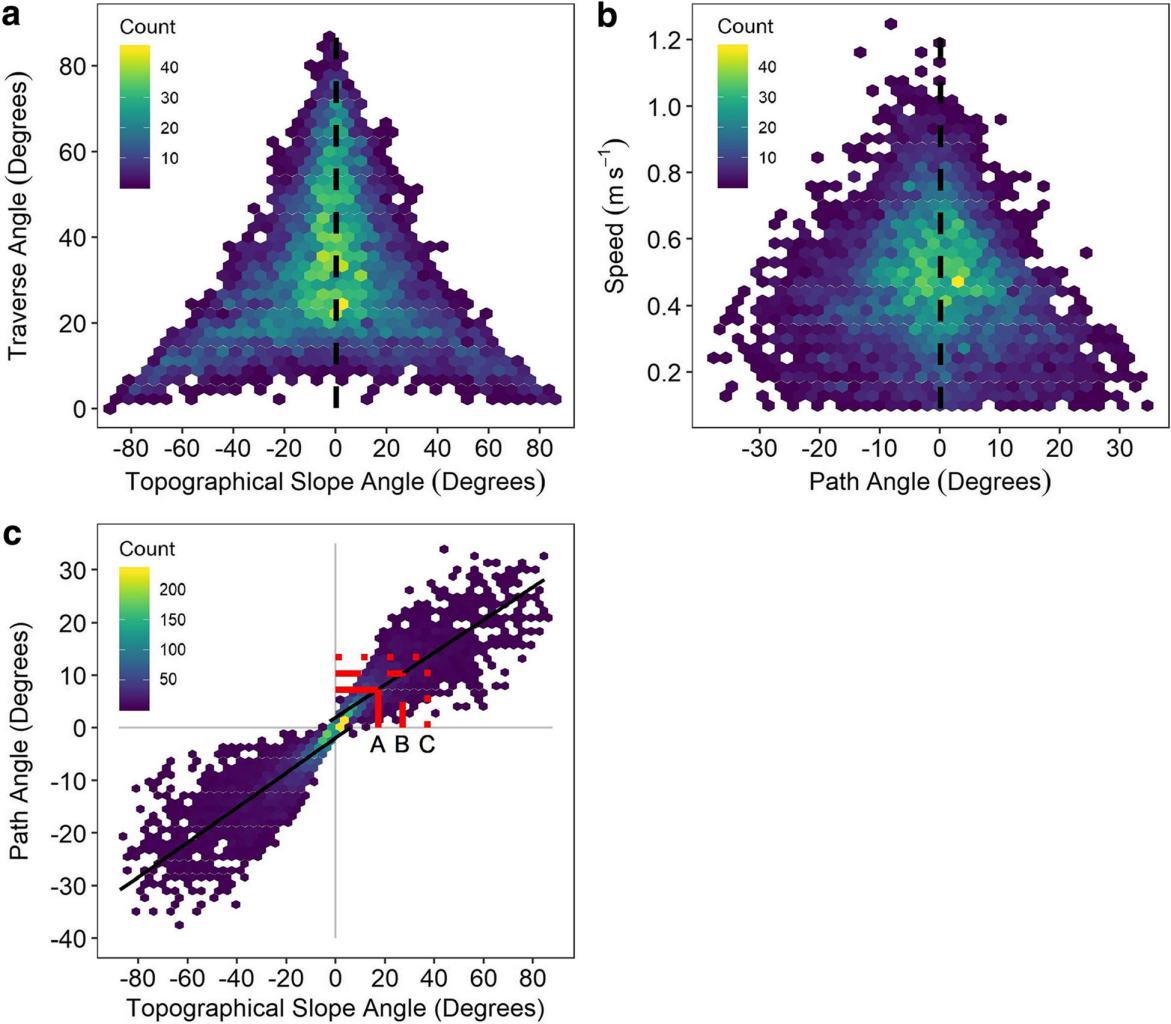
Density plots of (**a**) traverse angles in relation to topographical slope angle and (**b**) speed in relation to path angle and speed in relation to path angle. Positive path angles signify pumas walking on inclines. Negative path angles signify pumas walking on declines. The dashed line indicates level walking (0°). Colour scales represents the density of points (yellow = high density, blue = low density according to the ‘Count’ scale). (**c**): Density plot for topographical slope angle of the terrain in relation to path angle (selected incline by pumas). Least square regression lines are shown separately for incline and decline data (based on Eqs. 6 and 7). For incline data, the solid line A represents mean topographical slope angle and mean path angle. Dashed line B and dotted line C represent an extrapolation from the incline regression using a mean 10^o^ or 20^o^ increase in the topographical slope angle. The mean path angle would increase to 10.4^o^ and 13.5^o^ respectively. The colour represents density of points as described above

GPS location data were used to provide information on the distances and speed of travel, and the elevation change corresponding with that period. Puma home ranges were calculated as 95% minimum convex polygons (MCP) using a custom program based on the Python programming language (v. 2.7.9; Python Software Foundation, Wilmington, DE, USA). Elevation change was determined as the difference in elevation between sequential five-minute fixes where elevation was extracted for each location from an underlying digital elevation model (United States Geological Survey 2011). Similarly, horizontal distances travelled were determined as the distance between successive five-minute geo-location fixes. GPS-derived speed was calculated from distance travelled and time, and accelerometer-derived speed (in m s^− 1^) was calculated from ODBA (in *g*) using the relationship

1 $$ Speed=5.32\ast ODBA-0.42 $$

from Williams et al. [14]. During the observation period, pumas travelled through the mountainous landscape and climbed up and down varied slopes (Figs. 1 and 2). They could either travel up the slope of a hill directly, walking up the steepest possible angle of the hill, or they could traverse along the side of a hill at a shallower angle and thereby travel a longer distance. Pumas were assumed to travel in a straight line between sequential GPS recordings, and hence distances recorded were minimal distances travelled. To distinguish between the different paths chosen by pumas relative to the steepness of the hill, we used the terms, 1) GPS distance, 2) path distance, 3) elevation gain, 4) topographical slope angle, 5) path angle, and 6) traverse angle as defined by the angular deviation between compass headings of the path and the compass direction of the topographical slope (i.e. topographical aspect, Fig. 1a). Path distances travelled, and path angles taken were calculated geometrically. Topographical slope angles and path angles were either positive (inclining) or negative (declining); steep angles become more vertical, reaching +90^o^ (inclining) or -90^o^ (declining) and shallow angles are close to 0^o^. Traverse angles were determined so that values approaching 0^o^ represented travel perpendicular to topographical slope (i.e. cross-slope travel), and 90^o^ travel parallel to the direction of the slope (Fig. 1a).

### Behaviour identification

For each five-minute period, puma behaviour was classified into one of five categories, 1) resting, 2) non-mobile behaviours (e.g. feeding or grooming), 3) locomotion on an incline, 4) locomotion on a decline, and 5) unknown. Categorization was based on the GPS-derived speed and gradient that the animal was travelling and the relative activity, as recorded by the tri-axial accelerometer, in *g* (m s^− 2^) (see Supplementary Information).

### Calculating $\dot{\boldsymbol{V}}{\mathrm{O}}_2$ for pumas in the wild

The relationship between $\dot{V}{\mathrm{O}}_2$ while walking and surface gradient is described as linear and positive at slow speeds and shallow gradients [21, 33, 34]. Therefore, we assumed that the $\dot{V}{\mathrm{O}}_2$ of wild pumas increased linearly with the gradient of the angle they were climbing. To calculate puma $\dot{V}{\mathrm{O}}_2$ for inclines in the field, the difference between mean $\dot{V}{\mathrm{O}}_2$ on the level and on the 6.84^o^ treadmill incline was calculated for each of the speeds where data for both were obtained (0.56–1.11 m s^− 1^). The difference in $\dot{V}{\mathrm{O}}_2$ was then divided by 6.8^o^ (the treadmill angle) to give the $\dot{V}{\mathrm{O}}_2$ increase per one degree of incline. Hence, the $\dot{V}{\mathrm{O}}_2$ values of wild pumas traversing various mountain slopes were calculated as the $\dot{V}{\mathrm{O}}_2$ incurred when travelling on level ground plus the additional $\dot{V}{\mathrm{O}}_2$ incurred as a result of travelling on a particular incline. By extrapolation, we calculated the $\dot{V}{\mathrm{O}}_2$ of pumas walking on any angle at any speed (see Eq. 9) which was verified by comparison with other felids (see Supplementary Information).

In this study, declining path angles were assumed to result in a $\dot{V}{\mathrm{O}}_2$ cost similar to that of level locomotion. This is based on data reported by Raab et al. [35] in which the $\dot{V}{\mathrm{O}}_2$ values of dogs walking on the level or angles down to − 20.4° were not significantly different, and on the results of recent studies that have investigated the energetic cost of decline walking for other species [21].

The $\dot{V}{\mathrm{O}}_2$ (in mlO_2_kg^− 1^ min^− 1^) of wild pumas engaged in non-mobile activities (i.e., feeding, grooming) was calculated using ODBA (in *g*) as described in detail in Williams et al. [14] Eq. 2 where

2 $$ \dot{V}{\mathrm{O}}_2=58.42\ast ODBA+3.52 $$

$\dot{V}{\mathrm{O}}_2$ was converted to a whole-body field energetic cost (in kilojoules) by multiplying by 20.1 J ml^− 1^ and the puma’s mass (in kg) [36].

### Statistics

Analyses were conducted using R (version 3.4.0, R core team 2014), with a statistical significance level of *p* < 0.05 used. The results, unless otherwise indicated, are expressed as mean ± 1 standard error. General linear mixed (GLM) models were used to examine interactions between speed and treadmill angle on $\dot{V}{\mathrm{O}}_2$ (Supplementary Table 1). Each measurement on the treadmill was treated as an individual data point. GLM models were also used to determine the explanatory variables which best predicted traverse angle and speed during locomotion of wild puma on inclines. Likewise, GLM models were used to examine the interactions between topographical slope angle and traverse angle to explain path angle. Puma ID was included as a random factor. F-values were calculated using ANOVAs. Residuals of each model were examined for normality using QQplots.

## Results

### Oxygen consumption ($\dot{\boldsymbol{V}}{\mathrm{O}}_2$) during level and incline walking

**Fig. 3:**
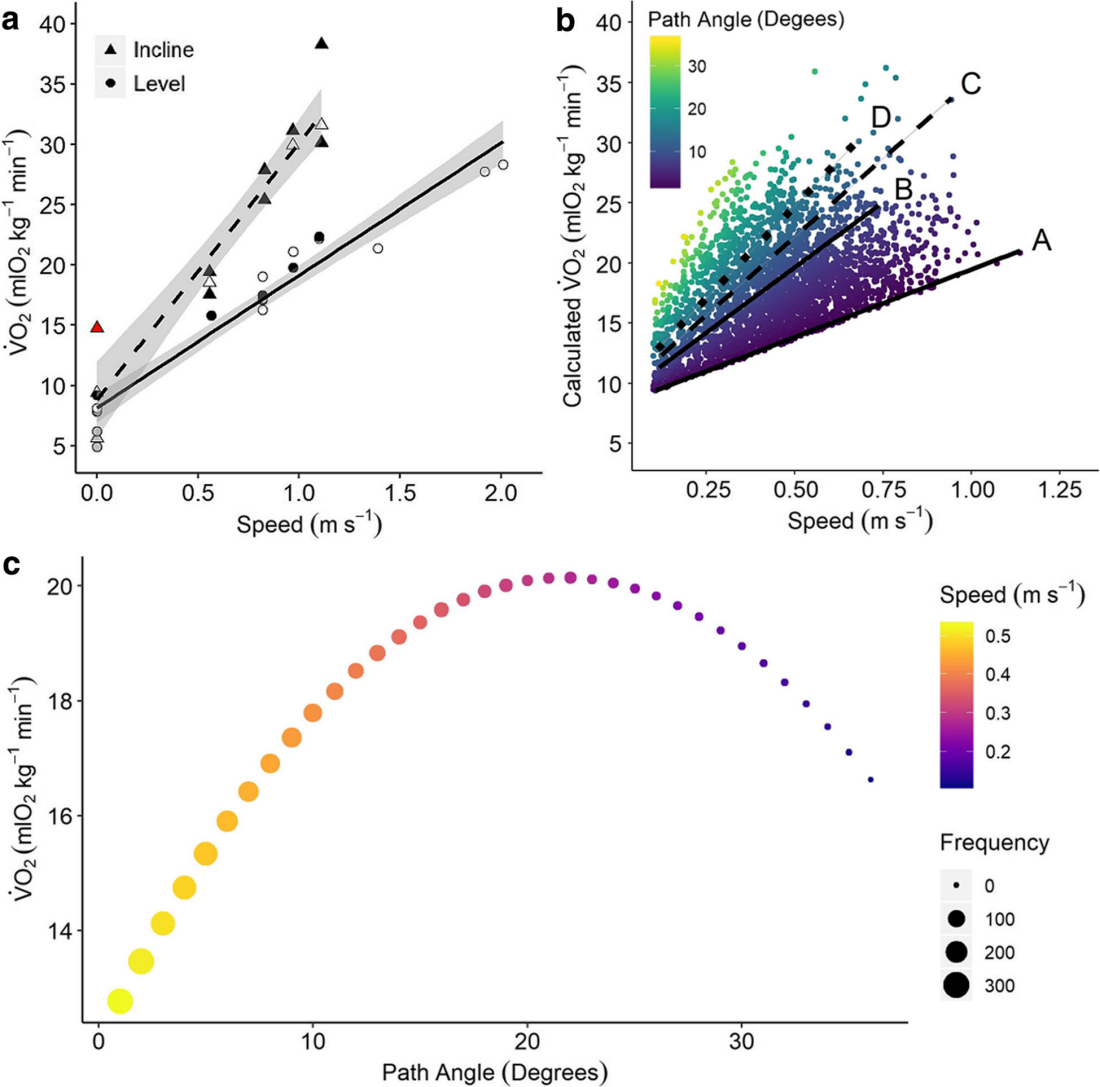
**a**: The rate of oxygen consumption ($\dot{V}$O_2_; mlO_2_kg^− 1^ min^− 1^) in relation to speed of pumas walking on a treadmill at an incline of 6.8° (*n* = 16, triangles, dashed line) and level walking at 0° (*n* = 20, circles, solid line). Point colour indicates individual pumas, two resting and locomoting on the treadmill (black, white), one resting only (grey) and one measurement where the ID was not noted (red). **b**: Calculated rate of oxygen consumption ($\dot{V}$O_2_; mlO_2_kg^− 1^ min^− 1^) in relation to speed for wild puma travelling on inclines. $\dot{V}$O_2_ was calculated from Eq. 9 using measured puma speeds and path angles. Path angle is represented by colour where yellow indicates pumas climbing up a steep path angle, and blue indicates pumas climbing a shallow path angle (see “Path Angle” scale). *N* = 2862 measurements of four wild pumas. Lines indicate $\dot{V}$O_2_ when walking on the level (A) and on the mean preferred path angle (B). The dashed and dotted lines indicate the predicted $\dot{V}$O_2_ if the mean topographical slope angle was to increase by 10° (C) or 20° (D) with increasing mean path angle accordingly. **c**: Modelled $\dot{V}$O_2_ (mlO_2_kg^− 1^ min^− 1^) for wild pumas from Eq. 9, using mean speeds of all recorded locomotion events at each path angle. Point size represents the frequency of use of each path angle by wild pumas. Values for $\dot{V}$O_2_ on inclines greater than 20 degrees should be interpreted with caution

The mean $\dot{V}{\mathrm{O}}_2$ of pumas at rest was 8.22 ± 1.09 mlO_2_kg^− 1^ min^− 1^, and increased with treadmill speed (in m s^− 1^) (Fig. 3a) where the least-squares fitted regression

3 $$ \dot{V}{\mathrm{O}}_2=10.99\ast speed+8.15 $$

explained 94.9% of the variation in $\dot{V}{\mathrm{O}}_2$ (F_1,18_ = 335.8, *p* < 0.001) (see also [14] Eq. 1). When pumas were walking at a 6.8^o^ incline, $\dot{V}{\mathrm{O}}_2$ also increased with higher treadmill speeds, at almost twice the rate. In this case, the resulting least-squares fitted regression

4 $$ \dot{V}{\mathrm{O}}_2=21.29\ast speed+8.83 $$

explained 90.6% of the variation in $\dot{V}{\mathrm{O}}_2$ (F_1,14_ = 134.2, *p* < 0.001). There was a significant interaction between speed and incline on $\dot{V}{\mathrm{O}}_2$ (*χ*^2^ =59.16, *p* < 0.001) such that $\dot{V}{\mathrm{O}}_2$ increased with speed at a faster rate when pumas were walking on an incline compared with when they were walking on the level. The highest $\dot{V}{\mathrm{O}}_2$ value recorded was 38.27 mlO_2_kg^− 1^ min^− 1^ when a puma was walking at its maximum voluntary speed (1.1 m s^− 1^) on the inclined treadmill.

The COT decreased with faster speeds (F_1,1_ = 124.87, *p* < 0.001), but increased with incline (F_1,1_ = 171.37, *p* < 0.001). There was no significant interaction between speed and incline on COT (F_1,1_ = 0.03, *p* = 0.85); COT decreased with speed at a similar rate during incline and level locomotion. The COT of pumas on the incline (0.15 ± 0.02 mlO_2_kg^− 1^m^− 1^) was 41.9 ± 7.20% greater than on the level.

### Wild puma behaviour

In total, 6037 five-minute windows of locomotory events were identified for the four pumas (mean per puma = 1509 ± 396 events, of which 715 ± 219 were incline events, and 795 ± 182 were decline events) (Supplementary Table 2). The four wild pumas spent a mean time travelling of 134.52 ± 8.30 min per day (9.3% of day). During this time, pumas spent 66.6 ± 4.6 min per day (4.6% of day) walking up inclining paths and 71.2 ± 4.0 min per day (5.0% of day) walking down declining paths. Non-mobile activities such as feeding and grooming accounted for 398.8 ± 8.7 min per day (27.7% of day). The pumas spent 851.4 ± 13.3 min per day (59.1% of day) resting. Unknown behaviours accounted for 37.7 ± 3.0 min per day (2.7 ± 0.2% of day). Individual pumas showed different ranging behaviours and home ranges (Supplementary Figure 2 and Supplementary Table 3).

Locomotory events identified for the four individuals indicated that the animals used a wide variety of terrains. The mean topographical slope angle encountered while climbing uphill was 17.2 ± 0.3^o^ (median = 11.0 ^o^, maximum = 84.6^o^). This compares with a mean decline topographical angle of − 19.1 ± 0.3^o^ (median = − 12.8^o^, minimum = − 87.5^o^) encountered while going downhill (Fig. 1c). During the study, pumas never climbed directly up steep slopes. Instead, they climbed steep slopes by traversing and choosing shallower path angles (mean inclining path angle 7.3 ± 0.1^o^, median = 5.5^o^). Pumas also avoided walking down steeply declining slopes directly (mean declining path angle − 8.3 ± 0.1^o^, median = − 6.3^o^). The maximum path angle for a climbing puma was 34.3^o^; the minimum for a descending puma was − 37.0^o^ (Fig. 1c).

During incline locomotion, there was a significant negative effect of topographical slope angle on traverse angle (*χ*^2^ = 1603.8, *p* < 0.001). Thus, as the topographical slope angle increased, the traverse angle and the path angle of the puma decreased (Fig. 2). Furthermore, there was a significant interaction between topographical slope angle and traverse angle on path angle (*χ*^2^ = 8182.4, *p* < 0.001). As pumas encountered progressively steeper slopes, they climbed at progressively shallower angles relative to the steepness of the slope. This strategy enabled pumas to avoid directly climbing up steep slopes (Fig. 1b). Consequently, 95% of path angles that pumas climbed were shallower than 19.74^o^. Last, there was a significant negative effect of path angle on the speed at which pumas travelled (*χ*^2^ = 521.4, *p* < 0.001), such that pumas travelled along shallower path angles at faster speeds and along steeper path angles at slower speeds (Fig. 2b).

Pumas also altered their behaviour during decline locomotion; as the topographical slope angle became more steeply downhill, traverse angle decreased (Fig. 2a). There was a significant positive effect of topographical slope angle on traverse angle (*χ*^2^ = 1879.4, *p* < 0.001) as well as a significant interaction between topographical slope angle and traverse angle on declining path angle (*χ*^2^ = 7489.5, *p* < 0.001). These findings suggest that as pumas encountered progressively steeper downhill gradients, they decreased the path angle steepness, thereby avoiding directly climbing down steep slopes. The speed of descent was affected, leading to a significant decrease in speed as declining path angle increased (*χ*^2^ = 230.41, *p* < 0.001). Thus, pumas travelled along shallower path angles at faster speeds and along steeper downhill path angles at slower speeds (Fig. 2b).

There was a positive relationship between path angle and topographical slope angle (Fig. 2c) as described by the least-squares regression

5 $$ Path\ Angle\ (degrees)=0.38\ast Topographical\ Slope\ Angle(degrees)-0.164 $$

This regression explained 87.4% of the variation in path angles (F_1,6032_ = 41,949.4, *p* < 0.001) when including both inclining and declining locomotion. There were, however, differences in the energetic cost of inclining and declining locomotion, and we cannot assume that pumas use the same path angles when travelling up and down the terrain. In view of this, the regressions are presented separately below. When pumas were travelling uphill only, the least-squares regression

6 $$ Inclining\ Path\ Angle\ (degrees)=0.31\ast Topographical\ Slope\ Angle(degrees)+1.98 $$

explained 74.3% of the variation in path angle (F_1,2857_ = 8303.1, *p* < 0.001). Similarly, when pumas were travelling downhill only, the least-squares regression

7 $$ Declining\ Path\ Angle\ (degrees)=0.33\ast Topographical\ Slope\ Angle(degrees)-1.94 $$

explained 75.1% of the variation in path angle (F_1,3170_ = 9735.1, *p* < 0.001). As might be expected due to increased options, there was more variation in the path angles chosen by pumas travelling on steeper inclines and declines. The range of path angles used was greatest at the topographical angles of − 60 degrees and 50 degrees (grouped by 10 degrees; Fig. 2c).

### Energy expenditure of wild pumas

In the wild, pumas encountered many different slope angles and climbed up and down many different path angles. For every degree of path angle incline above level travel, the additional increase in $\dot{V}{\mathrm{O}}_2$ with speed (m.s^− 1^) was

8 $$ \dot{\mathrm{V}}{\mathrm{O}}_2\  cost\  per\ 1\  degr\mathrm{e}e\  incline=\left(1.47\ast speed\right)+0.088 $$

(*n* = 4, r^2^ = 99.98). Based on this, the energetic cost of incline travel can be determined as: the regression for level $\dot{V}{\mathrm{O}}_2$ (Fig. 3a) plus the additional $\dot{V}{\mathrm{O}}_2$ cost per degree of incline multiplied by the path angle:

9 $$ \dot{V}{\mathrm{O}}_2\ \left( ml{\mathrm{O}}_2{kg}^{-1}{\mathit{\min}}^{-1}\right)=\left(8.15+10.99\ast speed\ \right)+\left( path\ angle\ast \left(1.47\ast speed+0.088\right)\right) $$

where path angle is in degrees and speed is in m s^−1^. Eq. 9 can be used to calculate the $\dot{V}{\mathrm{O}}_2$ of wild pumas during incline locomotion using GPS-derived speed and path angles. This determines $\dot{V}{\mathrm{O}}_2$ costs incurred across the range of speeds and path angles utilised by pumas (Fig. 3b).

We found that the maximum $\dot{V}{\mathrm{O}}_2$ during locomotion by a wild puma was 34.86 mlO_2_kg^−1^min^−1^, which occurred at a path angle of 16.4° and at a speed of 0.76 m s^−1^. The mean field $\dot{V}{\mathrm{O}}_2$ on inclines was 17.90 ± 0.08 mlO_2_kg^−1^ min^−1^, and the mean field $\dot{V}{\mathrm{O}}_2$ during declining locomotion was 14.08 ± 0.06 mlO_2_kg^−1^ min^−1^ (Table 1).

**Table 1:** Mean ± SEM rate of oxygen consumption ($\dot{V}$O_2_) and mean daily energy expenditure (DEE) of pumas in the Santa Cruz Mountains, calculated for daily behaviours. GPS-derived and accelerometery-derived speeds are compared. See Supplementary Table 4 for equations used to calculate energy costs. ‘*’ indicates that the values are the same for GPS- and accelerometery-derived $\dot{V}$O_2_ since these parameters are not based on speed

**Behaviour**	**GPS – derived speed**			**Accelerometery - derived speed**		
	$\dot{\boldsymbol{V}}$O_**2**_ (mlO_**2**_kg^**−1**^ min^**− 1**^)	$\dot{\boldsymbol{V}}$O_**2**_ (mlO_**2**_kg^**− 1**^ day^**− 1**^)	Energy (MJ day^**− 1**^)	$\dot{\boldsymbol{V}}$O_**2**_ (mlO_**2**_kg^**− 1**^ min^**− 1**^)	$\dot{\boldsymbol{V}}$O_**2**_ (mlO_**2**_kg^**− 1**^ day^**− 1**^)	Energy (MJ day^**− 1**^)
**Decline Locomotion**	14.08 ± 0.06	1007 ± 60.0	1.18 ± 0.07	25.04 ± 0.06	1802 ± 106.1	2.11 ± 0.12
**Incline Locomotion**	17.90 ± 0.08	1197 ± 84.9	1.40 ± 0.10	39.08 ± 0.28	2645 ± 190.6	3.09 ± 0.22
**Non-mobile Activity**	*15.23 ± 0.05	*6190 ± 128.0	*7.24 ± 0.15	*15.23 ± 0.05	*6190 ± 128.0	*7.24 ± 0.15
**Resting**	*8.22	*7101 ± 103.8	*8.30 ± 0.12	*8.22	*7101 ± 103.8	*8.30 ± 0.12
**Unknown**	*8.22	*310 ± 25.0	*0.36 ± 0.03	*8.22	*310 ± 25.0	*0.36 ± 0.03
**Total DEE**	–	15,640 ± 126.8	18.29 ± 0.15	–	17,661 ± 242.0	20.65 ± 0.28

DEE of the pumas depended on the behaviours exhibited on any given day. The mean DEE for the four wild pumas was 18.29 ± 0.15 MJ day^− 1^ (see Table 1 and calculations in Supplementary Information). Most of their time and energy was spent resting and on non-mobile behaviours (Supplementary Figure 3). Locomotion accounted for 13.97% of puma DEE, with incline travelling accounting for approximately half (7.58%) of this but only taking 4.65% of the total time. There were differences in the mean $\dot{V}{\mathrm{O}}_2$ of distinct behaviours (Table 1), leading to differences between time spent on the behaviour and the cost (Supplementary Figure 3); pumas spent more time on low-cost behaviours rather than costly activities. The calculated proportion of DEE used for locomotion increased to 24.64% when using accelerometer, rather than GPS-derived speed (Table 1). 

## Discussion

Energy landscape theory posits that animals generally avoid going through areas that pose a high energetic cost, and instead use a ‘path of least resistance’ strategy [13, 37]. As inhabitants of American deserts, the tropical flats of the Florida Everglades as well as the mountains of North and South America, pumas seem particularly adept at challenging this energetic optimization theory by living in a wide variety of habitats often considered as energetically demanding [13, 38]. This versatility, and the fact that approximately one third of existing puma habitat on private land in California will be lost by 2030 due to increased urban sprawl [39], makes this felid a particularly relevant animal model for investigating strategies for surviving in difficult landscapes. Here we find that several key behavioural modifications may enable pumas to circumvent some of the anticipated energetic costs of inhabiting steep terrains.

Locomotion on incline surfaces is known to be relatively costly for a wide variety of animals [21], including large mammals from dogs [35] and African lion cubs [33] to 440 kg thoroughbred racehorses [40]. As found in these previous studies, incline locomotion was more costly for pumas than level locomotion at comparable speeds with the difference in $\dot{V}{\mathrm{O}}_2$ for level and incline travel increasing progressively the faster the puma moved (Fig. 3a). Thus, the highest voluntary oxygen consumption rates on the treadmill (32–39 mlO_2_kg^− 1^min^− 1^) occurred at 2.0 m s^− 1^ during level running, and at a lower speed of 1.1 m s^− 1^ when on the incline.

Data from the SMART collars demonstrated how wild pumas avoided these excessive costs when traversing mountainous habitats. We found that pumas displayed a trade-off between speed, distance, and energy expenditure by modifying the chosen path of incline to affect the total cost of ascent (Figs. 1, 2 and 3). Through behavioural modifications, wild pumas could remain in an aerobic state during locomotor activity unless, for example, they were running at high speeds or on inclines during hunting. This is especially important for accommodating the relatively low aerobic scope of adult pumas (Fig. 3a [28]), where $\dot{V}{\mathrm{O}}_2$max is ~ 5 times resting levels rather than 6–10 times resting (at 49.2 mlO_2_kg^− 1^min^− 1^) predicted for quadrupeds of a similar body mass [41].

$\dot{V}{\mathrm{O}}_2$ costs of pumas were minimised by utilising two behavioural adaptations. First, wild pumas did not climb directly up inclines. Instead, they traversed steep slopes and thereby decreased the actual path angle climbed (Fig. 2), which lowered the rate of increase of $\dot{V}{\mathrm{O}}_2$ (Fig. 3b). Pumas decreased the mean path angle to 7.3^o^ from the mean topographical angle of 17.2^o^, and 95% of path angles were shallower than 19.74^o^. Second, wild pumas travelled slowly when they encountered steep slopes (Fig. 2b). The fastest speeds of 1.2 m s^− 1^ were seen around a path angle of 0^o^, while the speed on the steepest (> c.20^o^) path angles did not exceed 0.4 m s^− 1^ (Fig. 2b). The speeds most commonly used by pumas were slower than previously reported; we recorded a high density of points around 0.4–0.6 m s^− 1^ (Fig. 2b) for pumas in the wild. Previous estimates have been 1.1 m s^− 1^ for level locomotion by pumas in an enclosure [14]. Slower speeds required a higher $\dot{V}{\mathrm{O}}_2$ per meter travelled (i.e. higher COT) and travelling at very slow speeds would not be energetically efficient, despite requiring the lowest $\dot{V}{\mathrm{O}}_2$ per minute, as it would take a long time to travel any distance. Indeed, regularly-used human footpaths follow this trend, where the footpath does not take the least time to reachthe destination, but instead maximises the efficiency of metabolic cost for human locomotion [42]. There is also a trade-off on steeper slopes where faster speeds are presumably not energetically sustainable, so pumas utilised slower speeds [21, 33, 35].

Similarly, pumas travelling on steeply descending slopes selected shallow traverse angles. These shallower path angles were also travelled at faster speeds than when they moved down steeply descending path angles (Fig. 2). Pumas travelling down very steep slopes could be at risk of stumbling if they travel quickly [18]. Increased energy might be required to slow their descent on steep declines [21], however these were rarely used by wild pumas. The ‘bow tie’ shape of selected path angles in relation to topographical slope angle (Fig. 2c) shows that both inclining and declining path angles had very similar regression slopes, indicating that similar path angles were used during both ascent and descent. This would presumably occur if pumas followed the same paths up and down hillsides. Indeed, the selected path angles of pumas do minimise the cost of travelling (Table 2). Our results indicate that the preferred path for pumas involved traversing around hills rather than travel over them, as observed in other large mammals [24].

**Table 2:** Theoretical model for the rate of oxygen consumption ($\dot{V}{\mathrm{O}}_2$) based on a puma walking 100 m up a 30° path angle. Paths compared include climbing straight up or traversing back and forth at path angles of 2, 7, or 15 degrees. $\dot{V}{\mathrm{O}}_2$ is calculated per min and then totalled for how long it would take the puma to reach the end point based on the distance travelled and speed. ‘Poor’ energetic options are indicated due to the $\dot{V}{\mathrm{O}}_2$ exceeding maximum aerobic capacity or time exceeding 20 min. ‘Good’ energetic options show the optimal locomotion speed and path angle, and ‘moderate’ options are also indicated, which are often used

**Speed (m s**^**− 1**^**)**	**Path angle (degrees)**	$\dot{\boldsymbol{V}}$**O**_**2**_** (mlO**_**2**_**kg**^**− 1**^** min**^**− 1**^**)**	**Distance (m)**	**Time (min)**	**Total O**_**2**_** (mlO**_**2**_**kg**^**− 1**^**)**	**Energetic viability**
0.1	2.0	9.7	1432.7	238.8	2320.7	Poor
0.1	7.0	10.9	410.3	68.4	744.9	Poor
0.1	15.0	12.8	193.2	32.2	411.3	Poor
0.1	30.0	16.3	100.0	16.7	271.7	Moderate
0.5	2.0	15.3	1432.7	47.8	730.2	Poor
0.5	7.0	19.4	410.3	13.7	265.4	Moderate
0.5	15.0	26.0	193.2	6.4	167.4	Good
0.5	30.0	38.3	100.0	3.3	127.8	Poor
1.0	2.0	22.3	1432.7	23.9	531.4	Poor
1.0	7.0	30.0	410.3	6.8	205.5	Moderate
1.0	15.0	42.5	193.2	3.2	136.9	Poor
1.0	30.0	65.9	100.0	1.7	109.8	Poor

Presumably, travelling a further distance when traversing a hillside to avoid steep inclines provides an energetic benefit to the puma. To test this, we created a simplistic theoretical model examining energy use at various speeds and slopes (Table 2). Based on simple oxygen consumed, it initially appears that running up steep inclines at fast speeds would use less energy than traversing due to the short time it takes to reach the top. However, a key factor that must be considered in such calculations is the added cost of anaerobic metabolism when climbing quickly up steep inclines. Modelled rates of oxygen consumption exceeded measured puma $\dot{V}{\mathrm{O}}_2$ max for speeds greater than 0.5 m s^− 1^ when combined with the steepest path angles. Overall, the lowest calculated cost for climbing that did not exceed aerobic limits would occur at a modest speed of 0.5 m s^− 1^ with a path angle of 15°, which was similar to the observed movements of wild pumas (Fig. 2). Pumas in the wild may travel at modest speeds and shallower path angles to conserve energy, and to avoid entering anaerobic metabolism. Not surprisingly, anaerobically supported movements occur rarely for wild pumas except during brief bouts of prey capture (Fig. 2b; [14, 26]) and in this study, during high-intensity escapes from trailing hounds used for puma capture (Supplementary Information [26]). Pumas running up and down hills during these hound escape sequences far exceeded the maximal values of oxygen consumption observed during treadmill tests, and one puma expended > 3% of the mean puma DEE during a chase of less than 7 minutes (Supplementary Table 5). Thus, it is not surprising that short, steep uphill sprints are rare for wild pumas, despite the benefit of short travel time.

For wild pumas walking on inclines, there was a parabolic relationship between locomotion speed and duration which resulted in a trade-off between maintaining a low field COT and climbing incline terrains quickly (Fig. 3C). The lowest measured COT occurred during faster movements on the level treadmill; such paths and were commonly used by the cats in the wild. The steepest path angles also resulted in lower $\dot{V}{\mathrm{O}}_2$ costs compared with moderate incline angles performed at relatively fast speeds (Fig. 3c). This was due to the slow speeds used during these steep climbs and were rarely used by pumas.

Using these energetic data, we found that the calculated daily energy expenditure (DEE) of wild pumas, 18.29 MJ day^− 1^, living in a mountainous habitat was similar to that reported by Wilmers et al. [15] for wild African leopards (*Panthera pardus*) of 20.0 MJ day^− 1^. This could be a conservative estimate of puma DEE as they may have strayed from direct paths between the GPS coordinates or lost and gained altitude in this time. The accelerometer-derived speed would account for both of these but calibrations of ODBA on inclines would be needed to improve the accuracy of $\dot{V}{\mathrm{O}}_2$ calculations using this method.

Puma DEE values are higher than the allometric prediction for mammals of their body mass [43] (predicted 9.42 ± 0.27 MJ day^− 1^, t(4.3) = − 13.53, *p* < 0.001). There may be multiple reasons for this (see Supplementary Information). Although the mountainous landscape could lead to a high DEE despite behavioural strategies for minimizing energy costs, this figure is likely to be an overestimate, perhaps resulting from extrapolation of laboratory data and/or an elevated RMR due to pre-exercise anticipation and excitement [44]. Importantly, wild pumas spent less than 10% of the day locomoting and any incremental increase in energy expenditure due to inclines would increase the pumas’ DEE. The comparatively high DEE of the pumas in this study is also interesting as large carnivores are expected to maintain low energy expenditure due to unpredictable food sources [45]. The strategies seen here of pumas using predominantly slow speeds and shallow path angles indicate that they may be constrained by a low aerobic scope [28]. Pumas may use these strategies to ensure they do not fatigue or exceed their lactate threshold in steep terrains, both of which would prolong recovery times following exercise.

We can predict the impact of an increase in mean topographical slope angle by utilising the regressions above and therefore estimate the pumas’ DEE in steeper habitats. Using Eq. 6, one can infer that an increase in mean topographical slope angle by 10° or 20° would increase the mean path angle to 10.39^o^ or 13.48^o^ respectively. Assuming that the pumas would use the same speeds at these path angles as our study pumas, mean $\dot{V}{\mathrm{O}}_2$ (based on Eq. 9) would increase to 19.94 and 20.98 mlO_2_kg^− 1^ min^− 1^ for 10^o^ and 20^o^ slope increases, respectively. For the 20^o^ slope angle, this resulted in a 13% increase in $\dot{V}{\mathrm{O}}_2$ for incline locomotion resulting in an increase in daily locomotion energy costs from 1.40 MJ to 1.58 MJ per day; as a proportion of total DEE, the increase would be < 1.0%. It is important to note that steeper terrains could also increase $\dot{V}{\mathrm{O}}_2$ costs due to steeper declines; in this study we have assumed that decline costs are the same as for level locomotion, however, there are some cases where very steep declines are more costly than level locomotion [21, 46]. Another assumption of these calculations is that there is a linear relationship between incline and the rate of increase of $\dot{V}{\mathrm{O}}_2$ with speed. This may not be the case when pumas are travelling on the steepest slopes [21] and, while measurements of puma $\dot{V}{\mathrm{O}}_2$ on steeper treadmill inclines would have been interesting to collect, lack of these data does not detract significantly from our daily energy estimates because the wild pumas utilised steep slopes (above 20^o^) less than 5% of the time they were travelling. Nevertheless, $\dot{V}{\mathrm{O}}_2$ estimates for inclines much steeper than this should be interpreted with caution. Future models constructed from higher temporal resolution collars and ‘dead reckoning’ path reconstruction [47] may also help to further detail the energetic costs of steep terrain in relation to its associated impact on travel routes, path angles, distance, and the consequential behavioural choices made by pumas.

## Conclusion

This study investigated a single environmental challenge for pumas - incline locomotion - that must be considered in the context of other associated environmental factors that can affect overall energetic costs [19, 20]. Incline avoidance behaviours indicate a ‘path of least resistance’ strategy used by the pumas to decrease locomotion costs that will have an advantage through energetic conservation [48] especially in complex, costly habitats [14, 49]. Development of energy landscape modelling for pumas and other large carnivores could benefit from taking aspects of terrain - such as the steepness of slope - into account when identifying key habitats for conservation.

**Additional file 1.**

## Supplementary information

**Additional file 2.** Supplementary information

## Data Availability

All data on Dryad Puma data, Dryad, Dataset, 10.5061/dryad.4qrfj6q6w [50].

## Abbreviations

GPS: Global positioning system
DEE: Daily energy expenditure
$\dot{V}{\mathrm{O}}_2$: Rate of oxygen consumption
COT: Cost of transport
ODBA: Overall dynamic body acceleration
MCP: Minimum convex polygons
GLM: General linear mixed model
