# Supplementary material for: Surviving in steep terrain: a lab-to-field assessment of locomotor costs for wild mountain lions (Puma concolor)
Source: Mov Ecol. 2020 Aug 8;8:34. doi: 10.1186/s40462-020-00215-9 (PMC7414561; doi:10.1186/s40462-020-00215-9)
Supplement: Supplementary file 2 — Additional file 2. Supplementary information [file 40462_2020_215_MOESM2_ESM.docx]

Supplementary Information

**Behavioural Signature Library**

Identification of behaviours from accelerometer-signatures was calibrated with captive pumas (as per ref [1]). The pumas moved freely in their enclosure as well as participating in trained behaviours. Behavioural signatures were classified as resting, grooming, eating, drinking, pouncing, climbing, jumping, panting, and stalking as well as running on the treadmill and along a measured 20 m outdoor transect to include preferred walking, trotting and running gaits [1]. Behaviour categories that we considered to be important for the wild pumas were high and low acceleration movement (including running, trotting and walking), non-mobile activities (including eating and grooming), and resting. Kinematics (speed, stride frequency, stride length) were determined in captive pumas from a frame-by-frame analysis of video recordings using digital editing and motion analysis software (Corel Video Studio Pro 2x, Corel Corp., Ottawa, Ontario, Canada; ProAnalyst, Xcitex, Woburn, MA, USA). The data were compared for outdoor run sessions with the same pumas resting, walking and running on the treadmill.

**Open flow respirometry**

To measure the captive pumas’ oxygen consumption during locomotion, air was drawn through the metabolic chamber with a vacuum pump (FlowKit Controller, Sable Systems International, Inc., Las Vegas, NV) at 200 l.min^-1^ to maintain the fractional concentration of oxygen above 0.2000. Samples of air from the exhaust port were dried (Drierite) and scrubbed of carbon dioxide (Sodasorb) before entering a Sable Systems, Inc. oxygen analyser (Model FC-10). The percentage of oxygen in the expired air was monitored continuously and recorded once per second with a personal computer using Sable Systems Expedata Analysis software (Las Vegas, NV). Oxygen consumption was calculated using equations from Fedak *et al.* [2] and a respiratory quotient of 0.77 for mammalian carnivores [3]. All values were corrected to STPD. The entire system was calibrated daily with dry ambient air (20.94% O_2_) and every 3 to 4 days with dry N_2_ gas according to Fedak *et al.* [2] and Davis *et al.* [4].

**Behaviour Identification in the Field**

Uniquely identified behaviour categories from Williams *et al.* [1] were used to devise a decision tree to identify behaviours of wild pumas. Pumas were assumed to be resting when their GPS-derived speed was below 0.1 m s^-1^ and their average maximum overall dynamic body acceleration amplitude (ODBAa) was below 0.25 *g* for more than 80 % of the five-minute period. ODBAa was calculated by extracting the maximum ODBA amplitude over a sliding two-second window via Fast Fourier Transformation, which was then averaged over a sliding 15-second window to remove short-term variation and produce a representative value of dynamic acceleration relevant to the scale of GPS sampling. Animals were considered to be performing locomotory behaviours during these five-minute periods when their speed was greater than 0.1 m s^-1^ and when more than 80% of the time was spent undertaking ‘medium’, ‘high’ and ‘very high’ activity levels; for these behaviours, the ODBAa was greater than 0.25 *g* [5]. Where GPS fixes were not obtained every five-minutes (e.g. if the GPS logger failed to record a location), behaviour was defined as ‘unknown’. All other behaviours were classified as resting or ‘non-mobile’ activities, which could not be defined as ambulatory (See Supplementary Fig. 1).

**Evaluating the Accuracy of Incline Energetics Models for Felids**

Our quantification of the $\dot{V}$O_2_ of pumas walking on the inclining treadmill shows that there is a higher cost when on an incline than on the level, which increases at a linear rate with respect to speed (Fig 3A). The slope of the regression for level walking on the treadmill is 10.99, whereas the regression slope for incline walking at 6.8^o^ is 21.29. While we assume this will be most accurate when similar speeds and angles used on the treadmill are used by wild pumas, in fact, wild pumas did not exceed these speeds, and the mean path angle climbed was similar to the treadmill angle (7.3^o^ *versus* 6.8^o^, respectively).

To test the reliability of our equation for wild puma $\dot{V}$O_2_ during locomotion (Equation 9) we can compare known data with calculated $\dot{V}$O_2_ values of related species. Chassin *et al.* [6] published $\dot{V}$O_2_ values from indirect calorimetry for sub-adult lions on a treadmill at three angles: 0ᵒ, 12.6ᵒ, and 17.1ᵒ. We calculated the difference between the $\dot{V}$O_2_ measurements at 0ᵒ and at 17.1ᵒ for each speed and divide by 17.1, thus obtaining a regression for $\dot{V}$O_2_ increase per one-degree incline. Multiplying this value by 12.6 and adding it to the level regression (similar to Equation 9), we obtained predictions for $\dot{V}$O_2_ at a 12.6ᵒ incline. We then compared the calculated $\dot{V}$O_2_ values with the recorded values from Chassin *et al.* [6]; these were not significantly different (Mann-Whitney test, U = 3231, *p* = 0.87), which gives us confidence that equation 9 provides reliable estimates for $\dot{V}$O_2_ at the observed speeds and inclines.

**Assessing Daily Energy Expenditures in Felids**

The puma DEE found here was greater than the allometric prediction for mammalian DEE from Speakman and Krol [7] of 9.42 MJ day^-1^. In this regard, pumas were similar to African leopards where a field DEE of 20.0 MJ day^-1^ was found [5] but the allometric prediction for the DEE is 6.89 MJ day^-1^ for a mass of 36.4 kg. Scantlebury *et al.* [8] found the field DEE for wild cheetahs (*Acinonyx jubatus*) was 9.01 MJ day^-1^ using the doubly labelled water technique, and the predicted DEE would be 7.46 MJ day^-1^ (mean mass: 41 kg). The lower DEE of cheetahs compared to pumas could be due the former minimising energy costs in a desert, or alternatively, could be due to pumas requiring greater energy expenditure as they are live in a mountainous habitat. The calculated DEE of wild pumas was also measured for males that were walking long distances, presumably patrolling territories, and in addition they were travelling in a steep terrain, which will increase locomotory costs.

Another factor likely affecting the calculated DEE may be related to how speed is determined from the collar tags. The DEE measurements for pumas were determined using GPS-derived speed. $\dot{V}$O_2_ costs for locomotion are different if calculated using accelerometer-derived speeds (Fig. 4, Table 1). Calculations using the latter increases the DEE by 12.9% (up to 20.65 ± 0.28 MJ day^-1^), which is primarily due to the increase in the cost of incline locomotion from 7.58% to 14.64% of the DEE. Although the instantaneous speed measurement provided by accelerometer-derived data is potentially more accurate than assuming the puma travelled in a straight line between GPS coordinates, the path angles used for the calculations remain GPS-derived. If path angles could be calculated instantaneously, potentially through dead-reckoning [9,10], accelerometer-derived speeds and energy calculations could be improved.

Lastly, we can improve models for energy landscapes as our quantification of $\dot{V}$O_2_ during locomotion allows us to calculate sustained metabolic scope (SusMS; DEE/Daily resting metabolism; [11]) for pumas while taking the path angle into account [12]. Puma RMR measured during the respirometry data indicates a daily resting metabolism of 11837 mlO_2_.kg^-1^.day^-1^ (13.84 MJ day^-1^), leading to a SusMS of 1.32 x RMR. This is below the reported maximum of 6-7 x RMR which some mammals occasionally achieve [11,13] but not significantly different from allometric prediction of 1.39 (t(3)=2.32, *p* =0.10) for animals of a similar mass [14], and not significantly different to that calculated for cheetahs (1.55 ± 0.69, t(19.83) = -1.71, *p* = 0.10) [8]. The comparatively low SusMS of pumas could have been effected by a high measured RMR. Pre-exercise anticipation and excitement are known to increase RMR for some individuals, which would increase apparent resting $\dot{V}$O_2_ [15].

Supplementary References

(1) Williams TM, Wolfe L, Davis T, Kendall T, Richter B, Wang Y, *et al.* Mammalian energetics. Instantaneous energetics of puma kills reveal advantage of felid sneak attacks. Science 2014:346(6205):81-85

(2) Fedak MA, Rome L, Seeherman HJ. One-step N2-dilution technique for calibrating open-circuit VO2 measuring systems. J Appl Physiol Respir Environ Exerc Physiol 1981;51(3):772-776.

(3) Williams TM, Ben-David M, Noren S, Rutishauser M, McDonald K, Heyward W. Running energetics of the North American river otter: do short legs necessarily reduce efficiency on land? Comparative Biochemistry and Physiology Part A: Molecular & Integrative Physiology 2002;133(2):203-212.

(4) Davis R, Williams T, Kooyman G. Swimming metabolism of yearling and adult harbor seals Phoca vitulina. Physiol Zool 1985;58(5):590-596.

(5) Wilmers CC, Isbell LA, Suraci JP, Williams TM. Energetics‐informed behavioral states reveal the drive to kill in African leopards. Ecosphere 2017;8(6).

(6) Chassin PS, Taylor CR, Heglund NC, Seeherman HJ. Locomotion in lions: energetic cost and maximum aerobic capacity. Physiol Zool 1976:1-10.

(7) Speakman JR, Król E. Maximal heat dissipation capacity and hyperthermia risk: neglected key factors in the ecology of endotherms. J Anim Ecol 2010;79(4):726-746.

(8) Scantlebury DM, Mills MG, Wilson RP, Wilson JW, Mills ME, Durant SM, *et al.* Mammalian energetics. Flexible energetics of cheetah hunting strategies provide resistance against kleptoparasitism. Science 2014;346(6205):79-81.

(9) Bidder OR, Soresina M, Shepard EL, Halsey LG, Quintana F, Gómez-Laich A, *et al.* The need for speed: testing acceleration for estimating animal travel rates in terrestrial dead-reckoning systems. Zoology 2012;115(1):58-64.

(10) Dewhirst OP, Evans HK, Roskilly K, Harvey RJ, Hubel TY, Wilson AM. Improving the accuracy of estimates of animal path and travel distance using GPS drift‐corrected dead reckoning. Ecology and Evolution 2016;6(17):6210-6222.

(11) Peterson CC, Nagy KA, Diamond J. Sustained metabolic scope. Proc Natl Acad Sci U S A 1990;87(6):2324-2328.

(12) Wilson RP, Quintana F, Hobson VJ. Construction of energy landscapes can clarify the movement and distribution of foraging animals. Proc Biol Sci 2012;279(1730):975-980.

(13) Gorman ML, Mills MG, Raath JP, Speakman JR. High hunting costs make African wild dogs vulnerable to kleptoparasitism by hyaenas. Nature 1998;391(6666):479.

(14) Degen AA, Kam M. Scaling of field metabolic rate to basal metabolic rate ratio in homeotherms. Ecoscience 1995;2(1):48-54.

(15) Taylor, C.R., Schmidt- Nielsen, Raab, J. L. Scaling of energetic cost of running to body size in mammals. Am. J. Physiol. 1970; 219(4):1104-1107.

Supplementary Figures


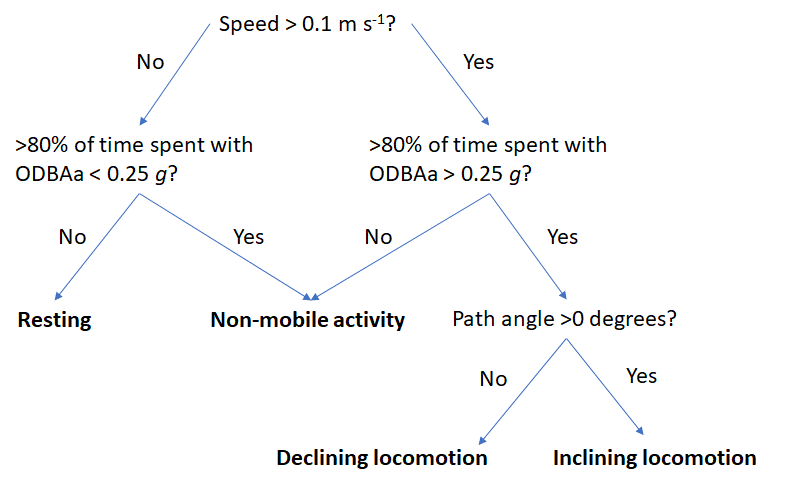


Supplementary Figure 1. Decision tree defining behaviours of wild pumas based on speed of travel and relative energy expenditure (ODBAa, *g*) obtained from the GPS and accelerometer loggers deployed on the puma collars.

**
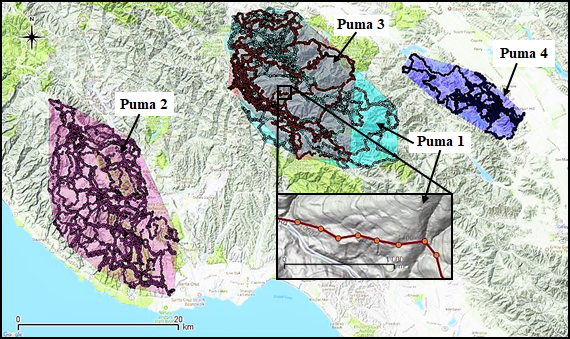
**

Supplementary Figure 2. Map of puma home ranges in the Santa Cruz Mountains, California: Puma 1 (turquoise), Puma 2 (purple), Puma 3 (maroon, overlapping with Puma 1, collared at different times), Puma 4 (blue). Inset shows an exploded map of a 40-minute section of incline locomotion from Puma 1, moving East. During this time Puma 1 travelled 1.042 km (mean speed 0.28 ms^-1^) and climbed 132.4 m in elevation; mean topographical slope angle was 12ᵒ. The pumas’ average path slope was 7.3ᵒ and the average traverse angle was 37.2ᵒ. Note contour lines on the hillside.

**
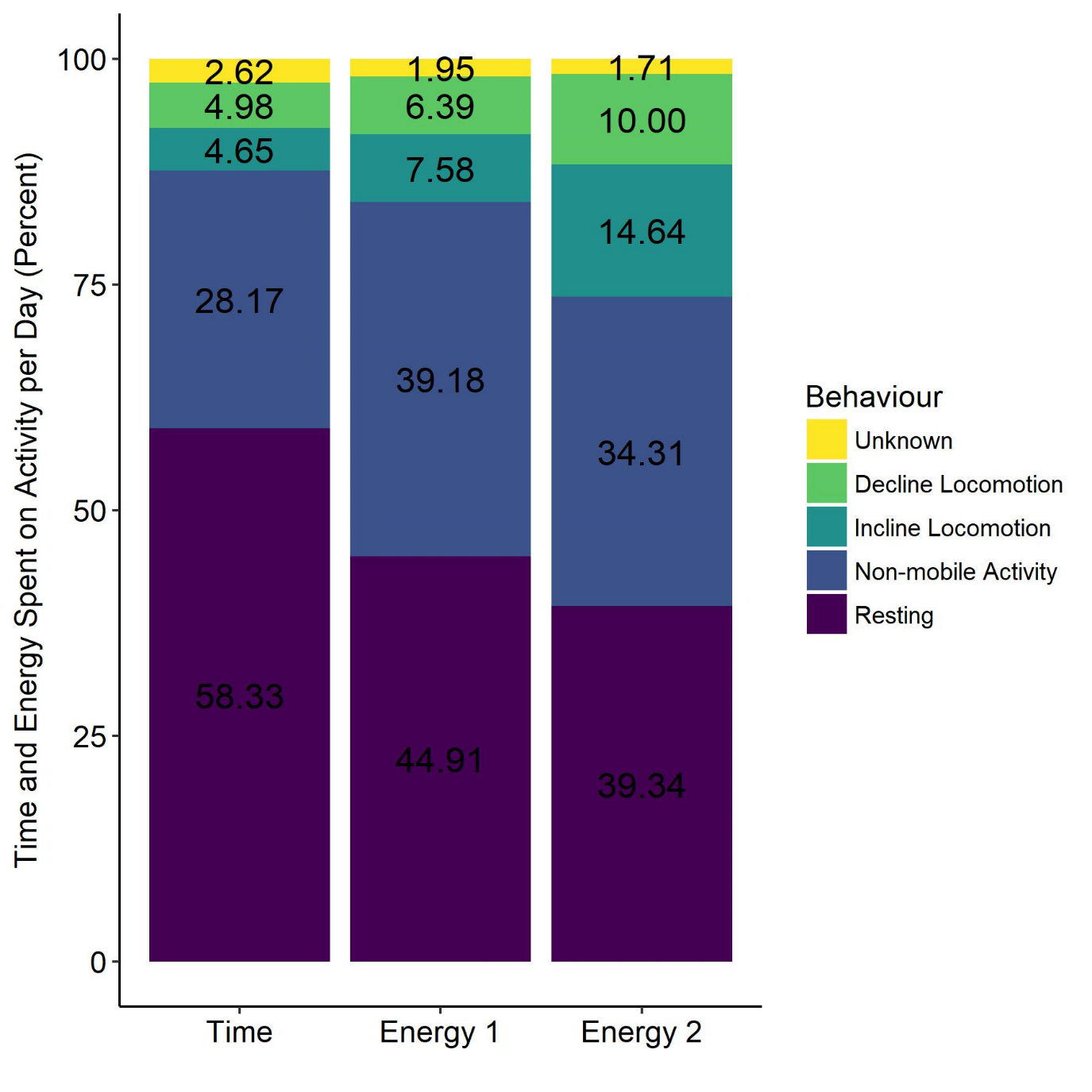
** Supplementary Figure 3. Time and energy allocation for pumas utilising different behavioural strategies by percent time resting (purple), non-mobile activity (blue), incline locomotion (aqua), decline locomotion (green), and unknown activities (yellow). Data were calculated using equations in Supplementary Table 4 for both GPS-derived (Energy 1) and accelerometer-derived (Energy 2) speeds.

| **Data used** | **Response** | **Explanatory** |
| --- | --- | --- |
| **Captive puma treadmill data** | $\dot{V}$O_2_ (mlO_2_kg^-1^min^-1^) | Treadmill angle*speed + 1\|puma ID |
|  | COT (mlO_2_kg^-1^m^-1^) | Treadmill angle*speed + 1\|puma ID |
|  |  |  |
| **Wild puma inclining locomotion data** | Traverse angle (Degrees) | Topographical slope angle + 1\|puma ID |
|  | Path angle (Degrees) | Topographical slope angle * traverse angle + 1\|puma ID |
|  | Speed (m s^-1^) | Path angle + 1\|puma ID |
|  |  |  |
| **Wild puma declining locomotion data** | Traverse angle (Degrees) | Topographical slope angle + 1\|puma ID |
|  | Path angle (Degrees) | Topographical slope angle * traverse angle + 1\|puma ID |
|  | Speed (m s^-1^) | Path angle + 1\|puma ID |

Supplementary Table 1. Response and explanatory variables of General linear mixed models. ‘*’ indicates inclusion of main effects and an interaction, ‘+’ indicates inclusion of main effects with no interaction. Puma ID was included as a random variable in all models, indicated as “1|”.

| **Puma ID** | **Age (yrs.)** | **Mass (kg)** | **Days collared** | **No. of**  **GPS fixes** | **Resting events** | **Non-mobile events** | **Incline locomotion events** | **Decline locomotion events** |
| --- | --- | --- | --- | --- | --- | --- | --- | --- |
| **1** | 7 | 50.8 | 60 | 17472 | 11074 | 4670 | 745 | 933 |
| **2** | 7 | 59.9 | 59 | 17197 | 8599 | 5858 | 1319 | 1240 |
| **3** | 6 | 62.1 | 51 | 14171 | 8709 | 4574 | 325 | 562 |
| **4** | 8 | 59.9 | 63 | 17504 | 12529 | 4063 | 473 | 440 |
| **Mean ± SE** |  | 58.2 ±2.51 | 58.3 ±2.56 | 16586 ±808 | 10228 ±956 | 4791 ±380 | 715 ±219 | 795 ±182 |

Supplementary Table 2. Details of GPS and locomotion classifications of wild pumas

Supplementary Table 3. Wild puma movement details with metrics on home ranges and locomotion behaviours (means ± SE).

| **Puma ID** | **Home range (km^2^)** | **Distance walked /day (km)** | **Elevation gain /day (m)** | **Incline path angle (Degrees)** | **Incline slope angle (Degrees)** | **Time on inclines /day (min)** | **Elevation loss /day (m)** | **Declining path angle (Degrees)** | **Declining slope angle (Degrees)** | **Time on declines /day (min)** |
| --- | --- | --- | --- | --- | --- | --- | --- | --- | --- | --- |
| **1** | 162 | 9.4  ±0.62 | 221.6 ±34.47 | 9.2  ±0.27 | 20.0 ±0.67 | 76.0 ±8.40 | -401.9 ±41.42 | -10.1 ±0.26 | -22.3 ±0.64 | 83.3 ±8.33 |
| **2** | 154 | 13.7 ±0.72 | 300.3 ±37.39 | 6.4  ±0.16 | 16.3 ±0.48 | 111.8 ±11.60 | -360.7 ±33.44 | -6.9 ±0.17 | -17.0 ±0.49 | 105.1 ±9.17 |
| **3** | 119 | 10.4 ±0.47 | 108.0 ±13.45 | 6.4  ±0.27 | 14.3 ±0.75 | 33.2 ±3.40 | -273.5 ±27.35 | -8.8 ±0.30 | -19.9 ±0.73 | 55.1 ±4.92 |
| **4** | 38 | 5.8  ±0.33 | 108.8 ±16.49 | 7.3  ±0.28 | 17.3 ±0.77 | 40.8 ±5.17 | -135.4 ±19.40 | -7.4 ±0.29 | -17.4 ±0.81 | 38.6 ±5.10 |
| **Mean ±SE** | 118  ±56.7 | 9.6  ±0.31 | 209.6 ±15.23 | 7.3  ±0.12 | 17.2 ±0.32 | 66.6 ±4.57 | -293.5 ±17.17 | -8.2 ±0.12 | -19.1 ±0.32 | 71.2 ±4.01 |

Supplementary Table 4. The equations used to calculate $\dot{V}$O_2_ (mlO_2_kg^-1^min^-1^) which are applied to wild puma behaviours. Speed is in m s^-1^, path angle is in degrees, and ODBA is in *g*.

| **Behaviour** | $\dot{\boldsymbol{V}}$**O_2_ calculation** |
| --- | --- |
| **Decline locomotion** | 10.99*speed+8.15 |
| **Incline locomotion** | (10.99*speed+8.15) + (path angle*(1.47*speed +0.088)) |
| **Non-mobile activity** | 58.42*ODBA+3.52 |

Supplementary Table 5. Summary of parameters for high-energy chases by pumas. Speeds are derived from accelerometers and path angles are derived from GPS elevation and distance (means ± SEM). $\dot{V}$O_2_ was calculated using Equation 3 for level and declining locomotion, and Equation 9 for inclining locomotion. Total energy cost of each chase is given in MJ and as a percent of the mean puma DEE.

|  | **Puma 1,**  **chase 1** | **Puma 1,**  **chase 2** | **Puma 2, chase 1** | **Puma 2,**  **chase 2** |
| --- | --- | --- | --- | --- |
| **Duration (seconds)** | 256 (4m 16s) | 58 (0m 58s) | 416 (6m 56s) | 168 (2m 48s) |
| **Path angle (degrees)** | -18.24 ± 4.71 | -9.54 ± 7.66 | 7.44 ± 1.04 | 10.58 ± 3.35 |
| **Speed (ms^-1^)** | 2.92 ± 0.32 | 11.52 ± 0.64 | 3.48 ± 0.43 | 3.53 ±0.47 |
| **Mean** $\dot{\boldsymbol{V}}$**O_2_**  **(mlO_2_kg^-1^min^-1^)** | 66.64 ± 15.83 | 174.48 ± 14.95 | 69.33 ± 8.07 | 139.71 ± 38.32 |
| **Total O_2_ (mlO_2_kg^-1^)** | 279.99 | 162.84 | 499.58 | 386.02 |
| **Total energy cost (MJ)** | 0.29 | 0.17 | 0.60 | 0.46 |
| **Total energy cost (% of mean DEE)** | 1.53 | 0.90 | 3.28 | 2.43 |

Supplementary Animation 1. Example path of a mountain lion (*Puma concolor*) travelling through the Santa Cruz Mountains (California, USA) showing how individuals do not attempt to climb steep inclines directly but instead traverse sideways at slower speeds. Path colours denote the speed of the puma, red is slow (<0.25 ms^-1^), yellow is intermediate (0.25-0.50 ms^-1^) and blue is fast speed (>0.50 ms^-1^).
